# 3D fabrication and characterization of phosphoric acid scaffold with a HA/*β*-TCP weight ratio of 60:40 for bone tissue engineering applications

**DOI:** 10.1371/journal.pone.0174870

**Published:** 2017-04-13

**Authors:** Yanen Wang, Kai Wang, Xinpei Li, Qinghua Wei, Weihong Chai, Shuzhi Wang, Yu Che, Tingli Lu, Bo Zhang

**Affiliations:** 1 Industry Engineering Department, School of Mechanical Engineering, Northwestern Polytechnical University, Xi’an, China; 2 Key Laboratory for Space Bioscience & Biotechnology, School of Life Sciences, Northwestern Polytechnical University, Xi’an, China; 3 Department of Urology, Tangdu Hospital, The Fourth Military Medical University, Xi’an, China; Kyoto Daigaku, JAPAN

## Abstract

A key requirement for three-dimensional printing (3-DP) at room temperature of medical implants depends on the availability of printable and biocompatible binder-powder systems. Different concentration polyvinyl alcohol (PVA) and phosphoric acid solutions were chosen as the binders to make the artificial stent biocompatible with sufficient compressive strength. In order to achieve an optimum balance between the bioceramic powder and binder solution, the biocompatibility and mechanical properties of these artificial stent samples were tested using two kinds of binder solutions. This study demonstrated the printable binder formulation at room temperature for the 3D artificial bone scaffolds. 0.6 wt% PVA solution was ejected easily via inkjet printing, with a supplementation of 0.25 wt% Tween 80 to reduce the surface tension of the polyvinyl alcohol solution. Compared with the polyvinyl alcohol scaffolds, the phosphoric acid scaffolds had better mechanical properties. Though both scaffolds supported the cell proliferation, the absorbance of the polyvinyl alcohol scaffolds was higher than that of the phosphoric acid scaffolds. The artificial stents with a hydroxyapatite/beta-tricalcium phosphate (HA/β-TCP) weight ratios of 60:40 depicted good biocompatibility for both scaffolds. Considering the scaffolds’ mechanical and biocompatible properties, the phosphoric acid scaffolds with a HA/β-TCP weight ratio of 60:40 may be the best combination for bone tissue engineering applications.

## 1 Introduction

Calcium phosphates are now used commonly in the medical field because of their chemical and structural similarities to the inorganic phase of human bone [1]. Hydroxyapatite (HA) is a major inorganic component of bone tissue, which has good bioactivity, biocompatibility and bone conductibility. However, pure HA is brittle and has slow degradation. β-tricalcium phosphate (β-TCP) has good biodegradability, biocompatibility, antibacterial and antimicrobial properties; conversely, β-TCP is of low mechanical strength and degrades too quickly in a physiological environment. The current hot topic in the study of biological materials is the blending of different biological materials together, in order to prepare high-performance composite materials and overcome the defects of a single component material [2]. Biphasic calcium phosphate (BCP), created by blending different biological materials, was introduced to overcome the shortcomings of single materials. This paper proposes that a proper composite of HA/β-TCP offers advantages for the use of calcium phosphate materials in tissue engineering.

Three-dimensional (3D) printing [3, 4] has been practised to fabricate calcium phosphate scaffolds. Advantages of 3D printing over traditional techniques comprise patient specific geometries and the incorporation of growth factor and drugs into the scaffolds. The feasibility of low temperature 3D printing of calcium phosphate scaffolds has been demonstrated [5–7]; this is a process in which the binder is used to join adjacent powder particles of the same and neighboring layers. Most literatures investigated on different concentrations of phosphoric acid solution to bond calcium phosphate powder [8]. In vivo and in vitro bone cells experiments, after all, these calcium phosphate scaffolds fabricated with acid binder solution need to regulate their pH value to neutral or slightly alkaline (pH 7.0–7.4). In addition, the acid binder solution can preclude the incorporation of growth factor and drugs into the scaffolds, and also cause damage of printhead and associated machine components. Elke Vorndran [9] concluded that the major challenge for the incorporation of living cells and growth factors in scaffolds during 3D printing is probably the chemical environment during setting, and alternatives may be the use of a binder which make cells and growth factors remain active during 3D printing process. Therefore a binder which can bond calcium phosphate powder at the room temperature and benefit the activity of the growth factors and drugs, should be developed. Polyvinyl alcohol (PVA) solution is beneficial to cells, bioactive molecules and drugs [10–13]. Many research utilize polyvinyl alcohol solution to fabricate calcium phosphate scaffolds by molding or paste injection instead of 3D printing [14]. Concerning calcium phosphate scaffolds, a limited number of powder-binder combinations has been demonstrated. Moreover, the phosphoric acid and polyvinyl alcohol exhibit chemical and physical binding mechanisms for binding calcium phosphate powder, respectively. It is necessary to investigate the printability of polyvinyl alcohol solution at room temperature 3D printing, and compare the effect of phosphoric acid and polyvinyl alcohol solution on the mechanical properties and biocompatible properties of calcium phosphate scaffolds.

The objective of this paper was to demonstrate the effect of HA/β-TCP composition ratios and two kinds of conventional biological binder solutions—phosphoric acid [8] and polyvinyl alcohol (PVA) [15]—on the mechanical properties and biocompatible properties of the 3D printed scaffolds, respectively. The proper powder-binder system, which can be applied at room temperature 3D printing and benefit incorporation of the bioactive molecules and drugs into the scaffolds, can be selected by comparing their physical and mechanical behaviors, as well as their biocompatibility. The hypothesis was that the composition of different binder solutions may improve the products accuracy, physical properties and biocompatibility of BCP scaffolds. Therefore, in this study, we fabricated two groups of scaffolds (phosphoric acid scaffolds and polyvinyl alcohol scaffolds) with four different ratios of HA/β-TCP (100/0, 20/80, 40/60, and 60/40).

## 2 Materials and methods

### 2.1 Powders and binder solution

For this study, we purchased the micrometer sized biomaterials HA and β-TCP powders (Bone Biological Technology Co., Ltd., Xi’an, China). HA powder is a composite of calcium phosphate phase with the Ca/P- ratio of 1.67. The Ca/P- ratio of β-TCP powder is 1.5. The appropriate amount of HA and β-TCP powders were weighed and mixed thoroughly to prepare the desired composition ratios of BCP (HA/β-TCP: 100/0, 20/80, 40/60, and 60/40). All reagents and chemicals were of analytical grade. PVA (MW 79156, Qingdao Usolf Chemical Technology CO., LTD, Shandong, China), phosphoric acid (Bai Shi Chemical CO., LTD, Tianjin, China, 85%), and Tween 80 (Hong Yan Chemical Reagent Factory, Tianjin, China,) were used. PVA solution (0.6, 0.8, 1 wt%) was prepared by heating at 95°C and after cooling to room temperature; phosphoric acid (8.75 wt%) [8] was prepared using deionized water to dilute the phosphoric acid (85 wt%). Tween 80, a non-cytotoxic surfactant, was added to PVA solution (0.6, 0.8, 1 wt%) and phosphoric acid (8.75 wt%) at a concentration of 0.25 wt% to reduce binder solution’s surface tension. By comparing the physical, mechanical and biological properties of the bone scaffolds, we selected a suitable powder–binder system.

Considering that binder properties affect the printability of a solution via inkjets, the surface tension, viscosity, and density of the binder solutions were measured. The viscosities were measured using a rotational viscometer (DV-1, Genggeng Instruments Equipment Co., Ltd. Shanghai, China) at 25°C and a shear rate of 100 s-1.The binder surface tensions were measured on a fully automatic surface tensionmeter (BZY-1,Pingxuan Scientific Instruments Co., Ltd. Shanghai, China) at 20°C.

### 2.2 Fabrication of scaffolds

Macroporous scaffolds (height = 3 mm, diameter = 10 mm and porosity = 50% with macropores of 800 μm) were designed using Solidworks CAD software (Dassault Systèmes SolidWorks Corp, USA) and converted to the standard file format for STL. A ZPrinter 250 printer (ZCorporation Inc., USA; now owned by 3D Systems Inc., USA) was then used to print the designed macroporous scaffolds using a powder layer thickness of 0.1 mm and binder spray of 0.3 L/m^2^. After they were built, the scaffolds were left to dry at 50°C for 2 h, as recommended by the printer manufacturer, before being removed from the build bed. De-powdering was performed using compressed air directed through a syringe needle.

Two main groups of BCP scaffolds, polyvinyl alcohol scaffolds (P) and phosphoric acid scaffolds (H), were fabricated using a 3D printer. Four different composition ratios of HA/β-TCP were produced within each group. Table 1 cites the study groups and fabricated BCP scaffolds ratios.

**Table 1:** Study groups and fabricated biphasic calcium phosphate scaffolds ratios.

Group	HA/β-TCP % Ratio	Logogram
**Polyvinyl alcohol**	100/0	P100
	20/80	P2080
	40/60	P4060
	60/40	P6040
**Phosphoric acid**	100/0	H100
	20/80	H2080
	40/60	H4060
	60/40	H6040

### 2.3 Measurement of physical properties of scaffolds

#### 2.3.1 Materials phase analysis for bone scaffolds

XRD was carried out using an X-ray diffractometer (X'Pert PRO, PANalytical, the Netherlands) with Cu-Kα (λ = 0.15418 nm) incident radiation. Phase of calcium phosphate powders and 3D bone scaffolds were qualitatively evaluated by X-ray diffraction spectra. Data collection was performed in the range of 2θ = 20–40° with a step size of 0.033°.

#### 2.3.2 Microstructures of BCP scaffolds

Micro morphology of scaffolds includes pore configuration, pore connectivity and pore distribution, etc. It has a direct effect on the bioactivity of scaffolds, the adherence of cells to scaffolds, the exchange of substances and so on. In this paper, the micro morphology of scaffolds was obtained using a scanning electron microscope (VEGA 3 LMU, OXFORD Instrument, UK) at different levels of magnification. The printed scaffolds were sputtered with gold (e-1045, HITACHI, Japan).

The scaffolds were placed on the bed of the high-resolution micro-CT scanner (Inveon, Siemens, Germany). The scaffolds were reconstructed three-dimensionally using software Mimics 10.01 (Materialise, Leuven, Belgium).

#### 2.3.3 Hydrophilicity of BCP scaffolds

The hydrophilic property of a cell has an obvious influence on the adhesion to its holder: the better the hydrophilicity, cells easily adhere to scaffolds; this is crucial, because only by being effectively adhered to the scaffolds can cellular migration, proliferation and differentiation proceed. [16, 17] This study evaluates the stents hydrophilic rate by contact angle and the bibulous rate. Bibulous rate measurement is obtained from the data recorded by measuring the weight of the stents noted by the microbalance at certain intervals; the sample is placed in deionized water, and then the water absorption is calculated. The bibulous rate formula [18] is as follows:

$$Bibulous\;rate=\frac{W_{2}-W_{1}}{W_{1}}$$ (1)

*W*_*1*_ is the net weight of support; *W*_*2*_ is the quality of the support measured each time.

In order to investigate the influence of water solution on the scaffolds’ dimensions, the dimensional variation of the scaffolds was calculated by comparing the physical dimensions of the scaffolds before and after measuring bibulous rate.

#### 2.3.4 Porosity of BCP scaffolds

The appropriate porosity is favorable to cellular adhesion, proliferation, blood vessels and nerves ingrowth and exchange of nutrient and metabolic waste. Briefly, porosity of a scaffold was calculated according to the following equation

$$\text{Porosity}=\left(1-\rho_{a}/\rho_{t}\right)\times 100\text{\%}$$ (2)

*ρ*_*a*_, the apparent density of the dried scaffolds, is calculated according to the Archimedes principle with scaffolds being immersed in mercury (Hg) solution. *ρ*_*t*_ is the theoretical density of the BCP scaffolds. The theoretical density of HA and β-TCP is 3.156 g/cm^3^ and 3.07 g/cm^3^, respectively. [19]

In addition, the porosity of the 3D printed scaffolds was analyzed using an AutoPore IV 9500 mercury porosimeter (Micromeritics, UK) at pressure in the range between 3.7 kPa and 414 MPa, in order to compare with the porosity measured by the aforementioned method.

#### 2.3.5 Biomechanical properties of BCP scaffolds

Scaffolds implanted in the human body for bone defect repair must have a certain mechanical strength, and this research aimed to test scaffolds’ compressive strength using the Universal Testing Machine.

The scaffolds with 15 mm diameter and 15 mm height were prepared for the mechanical characterization. Using the Universal Testing Machine (CTM250, Xieqiang Instrument Manufacturing (Shanghai) Co.,Ltd. Shanghai, China) with a 1kN load cell. The printed scaffolds were subjected to an axial pressure at a constant cross head speed of 2 mm min^−1^. Six of such measurements were conducted for each scaffold.

Different mechanical parameters can be obtained via stress–strain curves. For example, the compressive strength is the capacity of a structure to withstand loads that tend to reduce size; the tangent modulus is the slope of the compressive stress–strain curve.

In addition, the pure HA phosphoric acid scaffolds with diameter of 15 mm, height of 15 mm, macropore size of 1200 μm and porosity of 0%, 30% and 50% were fabricated in order to study the effect of porosity on the mechanical properties of these artificial bone scaffolds.

### 2.4 In vitro cell activity and infiltration

New Zealand rabbits (1 month old, male) were purchased from the Experimental Animal Center of the Fourth Military Medical University [Animal License No.: SCXK-(Militar) 2012–007]. Rabbits were sacrificed by overdose injection of sodium pentobarbital. Bone marrow (BM) was harvested from the tibia and femur condyle under aseptic conditions. The BM-PBS mixture (10 mL) was centrifuged for 8 min at 1,000 rpm (L500, XiangYi, China), and the supernatant was removed. Pellets were suspended in cell culture medium that contained α-minimum essential media (α-MEM) (Gibco, USA), 10% fetal bovine serum (FBS) (Gibco, USA), and 2% penicillin/streptomycin/amphotericin B (Sangon Biotech, China) and then centrifuged again. Then, the cell sediment was re-suspended in a 10 mL cell culture medium and cultured in 5% CO_2_ at 37°C and 95% relative humidity. BMSCs were obtained by complete medium changes after 2 days. Following this, the culture medium was changed every 3 days. After reaching approximately 85% confluence, the BMSCs were trypsinized with 0.25% trypsin and counted to yield the primary passage (P0). BMSCs (passage 0) continued to passage every 3 days, and then early passage BMSCs (passage 3) were used in the experiments.

The scaffolds with 5 mm diameter and 5 mm height were formed for biocompatible experiments. The eight kinds of scaffolds samples were neutralized to pH 7 with NaOH and sterilized by being soaked in 100% ethanol for 24 h and rehydrated in sterile PBS overnight. The scaffolds were placed in 24-well plates. BMSCs were seeded on scaffolds at an initial density of 1 × 10^5^ cells/well and given 7–10 days to adhere and establish themselves in the scaffolds in 5% CO_2_ at 37°C and 95% relative humidity. BMSCs were cultivated for 14 days in osteogenic medium, which included α-MEM supplemented with 5% FBS, 50 mM l-ascorbic acid, 0.1 mM dexamethasone, 1% antibiotic/antimycotic and 10 mM β-glycerophosphate. The culture medium was changed every 1 day.

The proliferation of BMSCs cultured on eight kinds of scaffolds was determined by the Cell Counting Kit-8 (CCK-8, MP Biomedicals, USA). 1 × 10^5^ cells/well were seeded onto each scaffold and cultured with regular α-MEM medium in 24-well plates. On D3, D7 and D14, the culture medium was changed to refresh the α-MEM medium with 10% CCK-8 solution. An aliquot from each well was transferred to a fresh 96-well plate. The light absorbance was measured at 450 nm using a microplate reader PowerWave XS (Bio-Tek, USA).

The cell-seeded scaffolds were washed with PBS and fixed in 2.5% glutaraldehyde. Following dehydration through a series of graded ethanol (5%, 10%, 25%, 50%, 75%, 90%, 95%, and 100%), the scaffolds were dried at room temperature for 24 h. The cell-seeded scaffolds were coated with gold (e-1045, HITACHI, Japan) and then imaged in an SEM (S-4800, HITACHI, Japan).

### 2.5 Statistical analysis

According to the SPSS 13.0 statistical software package, we analyzed the date. The experimental results were noted in the form of average value±standard deviation ($\tilde{x}\pm s$), and statistical results were analyzed by variance and examined by q. A value of p<0.05 means there was statistical significance. Mechanical tensile tests were performed with n = 6 samples per group, biomolecular functionalization experiments were performed with n = 3 samples per group, and all cell experiments were performed with n = 6 samples per group.

## 3 Results and discussion

### 3.1 Optimization of binder solutions

The density, viscosity, surface tension of the binder solutions were measured (Table 2). Supplementing the binder with 0.25 wt% Tween 80 did reduce the solution’s surface tension which met the demand of the 3D printing binder [20]. However, the viscosity of the binder did not improve. Through six printing experiments, those binders (1 wt% PVA+0.25 wt% Tween 80, 0.8 wt% PVA+0.25 wt% Tween 80) were difficult to be ejected fluently. The main reason was that 1 wt% PVA+0.25 wt% Tween 80 and 0.8 wt% PVA+0.25 wt% Tween 80 possessed higher viscosity which is unsuitable for being used as 3D printing binders. Therefore, 0.6 wt% PVA+ 0.25 wt% Tween 80 and 8.75 wt% phosphoric acid +0.25 wt% Tween 80 were selected for all subsequent experiments.

**Table 2:** Fluid characteristics of binder solution.

Binder	Tween 80 (wt %)	Density(g/ml)	Surface tension (mN/m)	Viscosity(mPa.s)
**8.75 wt% phosphoric acid**	0	1.05±0.03	67.9±0.5	1.05±0.04
**8.75 wt% phosphoric acid**	0.25	1.06±0.02	47.5±0.7	1.06±0.08
**1 wt% PVA**	0	1.06±0.02	84.9±0.9	1.74±0.10
**1 wt% PVA**	0.25	1.00±0.04	45.4±0.5	1.67±0.11
**0.8 wt% PVA**	0	0.96±0.02	70.5±0.6	1.37±0.03
**0.8 wt% PVA**	0.25	0.99±0.03	45.5±0.8	1.45±0.03
**0.6 wt% PVA**	0	0.96±0.03	53.1±0.9	1.12±0.02
**0.6 wt% PVA**	0.25	0.98±0.03	46.9±0.6	1.19±0.02

### 3.2 The prepared scaffolds

**Fig 1:**
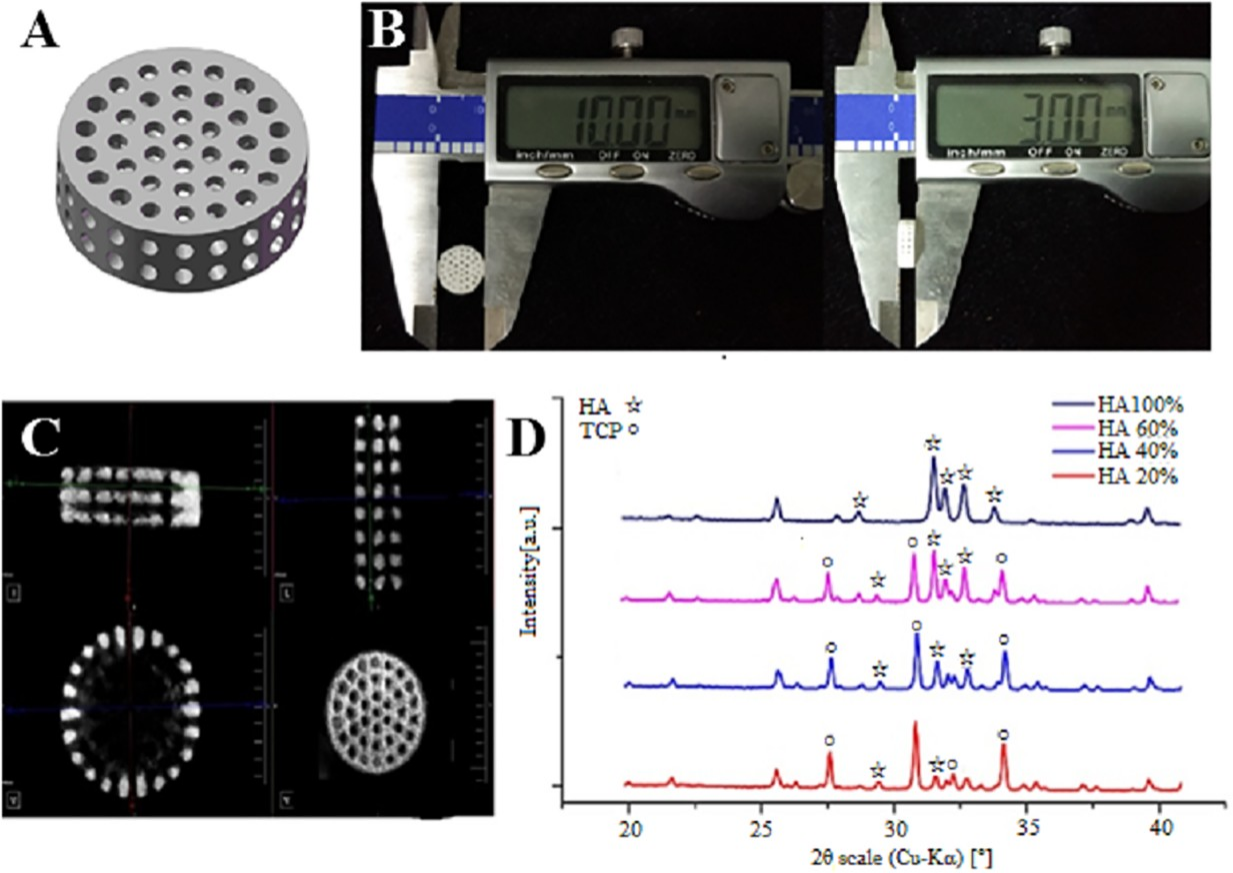
Characteristics of 3D printed different composite ratios of HA/β-TCP scaffolds with 8.75 wt% phosphoric acid solution. (A) Designed scaffolds using Solidworks CAD software. (B) The structural dimensions of the scaffolds. (C) Micro-CT and 3D reconstruction images of the scaffolds. (D) XRD pattern of the scaffolds with different HA/β-TCP ratios.

The cylindrical scaffolds with a diameter of 10 mm, a height of 3 mm, a macropore size of 800 μm and porosity of 50% were designed using Solidworks CAD software (Fig 1A). Fig 1B shows the structural dimensions of the scaffolds. The diameter and height of the prepared scaffolds were consistent with the geometrical model, and they were Φ10mm and H3mm, respectively. Micro-CT and 3D reconstruction images showed layers of powders, macropores and the struts of the scaffolds (Fig 1C).

The results of XRD analysis are shown in Fig 1D. The XRD patterns of pure HA and β-TCP scaffolds matched well with those of standard phase-pure HA (XRD JCPDS file (No. 09–0432) and β-TCP (No. 09–0169), respectively. The varying intensities and different peaks showed the gradual variance in the phase composition of the scaffolds with different percentages of HA/β-TCP weight.

### 3.3 The analysis of the properties of BCP scaffolds

#### 3.3.1 Hydrophilicity of scaffolds

**Fig 2:**
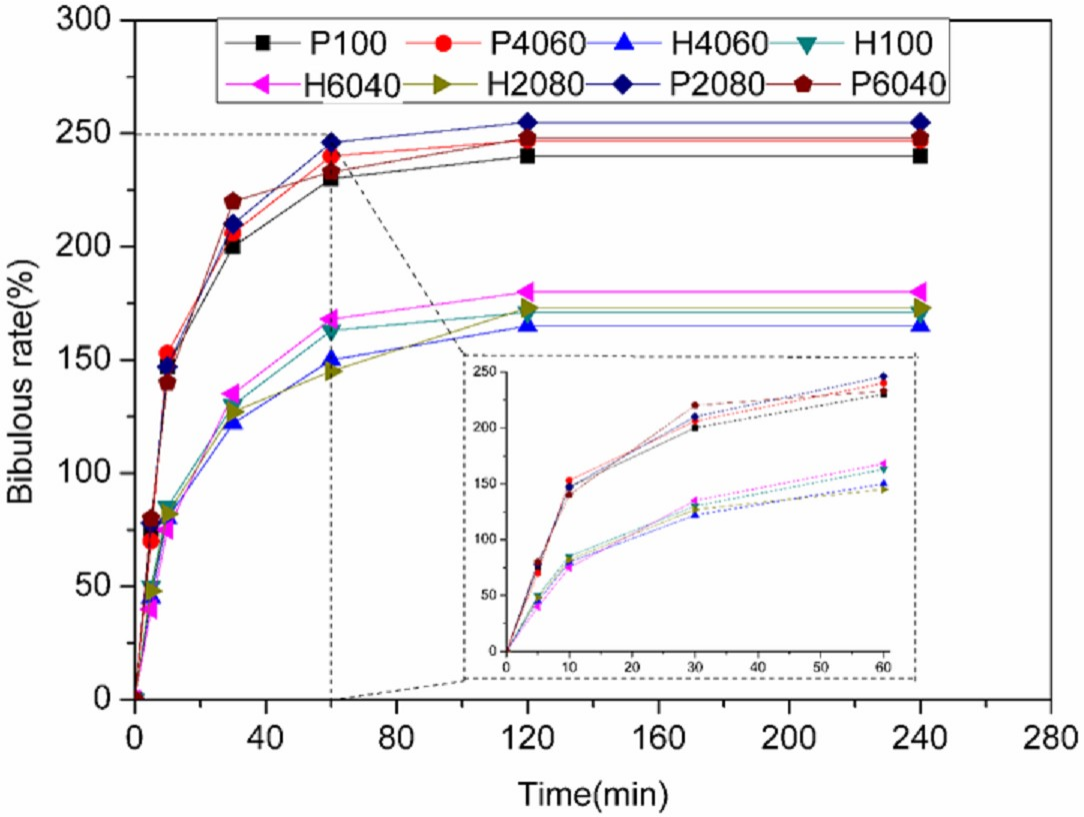
Bibulous rate of the printed scaffolds (0.6 wt% polyvinyl alcohol + 0.25 wt% Tween 80 (P) and with 8.75 wt% phosphoric acid + 0.25 wt% Tween 80 (H)) with different HA/β-TCP ratios.

The weights of eight kinds of scaffolds were recorded in a specific sequence of time (min), calculating the water absorption rates according to the above bibulous rate formula. The calculated water absorption rates are shown in Fig 2. For both the phosphoric acid scaffolds and the polyvinyl alcohol scaffolds, the HA/β-TCP ratios had no effect on hydrophilicity. For the phosphoric acid scaffolds, the average water absorption rate was 173%, while the average water absorption rate of the polyvinyl alcohol scaffolds was 248%.

Apparently, composition has no significant influence on hydrophilicity. However, there is a significant difference between the phosphoric acid scaffolds and the polyvinyl alcohol scaffolds in hydrophilicity. Compared with the phosphoric acid scaffolds, the polyvinyl alcohol scaffolds have a higher hydrophilic property. This is because the PVA proves to have more hydrophilicity.

Scaffolds’ dimensions were analyzed to evaluate the influence of water solution. This information plays a pivotal role in customized implants. The geometrical variations are listed in Table 3. Obviously, the dimensional variations of the polyvinyl alcohol scaffolds are higher than those of the phosphoric acid scaffolds, which shows that the polyvinyl alcohol scaffolds have better hydrophilicity. However, the polyvinyl alcohol scaffolds possess higher dimensional variations while encountering with water solution, which reveals that the shape of scaffolds deformation is larger than 5%. It is hard to meet the requirements of most bone implants in clinical surgery.

**Table 3:** Dimensional variations of the scaffolds before and after measuring bibulous rate.

Scaffold	Dimensional variation in diameter (%)	Dimensional variation in height (%)
**P100**	5.61±0.15	6.5±0.2
**P2080**	5.8±0.12	7.10±0.12
**P4060**	6.01±0.12	6.2±0.11
**P6040**	5.59±0.13	6.6±0.17
**H100**	2.01±0.09	1.86±0.12
**H2080**	1.93±0.11	1.88±0.21
**H4060**	1.95±0.08	1.53±0.16
**H6040**	1.75±0.20	1.68±0.09

#### 3.3.2 Mechanical properties of scaffolds

**Fig 3:**
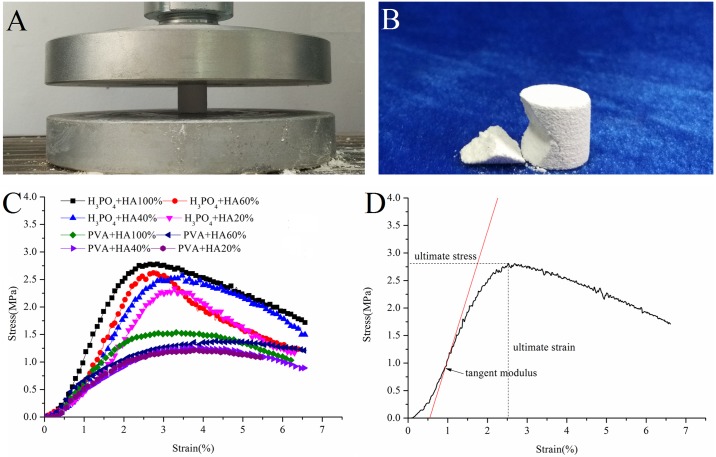
(A) Mechanical properties test. (B) Fragment of bone scaffolds. (C) A stress–strain curve of the printed scaffolds (polyvinyl alcohol scaffolds (PVA) and phosphoric acid scaffolds (H_3_PO_4_)) with different HA/β-TCP ratios. (D) A representation of the compressive stress, corresponding strain and tangent modulus.

Compression tests were performed to characterize the mechanical properties of the prepared scaffolds. Fig 3C shows stress–strain curves for the scaffolds printed. Fig 3D is a representation of the compressive stress, corresponding strain and tangent modulus. The summary of mechanical properties is reported in Table 4.

**Table 4:** Mechanical properties of produced BCP scaffolds.

Group	Compressive strength (MPa)	Tangent modulus (MPa)	Strain (%)
**PVA+HA100%**	1.54±0.13	70±4	2.5±0.12
**PVA+HA20%**	1.21±0.11	46±3	3.2±0.17
**PVA+HA40%**	1.26±0.09	44±3	3.4±0.14
**PVA+HA60%**	1.35±0.11	38±2	2.8±0.18
**H**_**3**_**PO**_**4**_**+HA100%**	2.81±0.08	147±10	3.0±0.12
**H**_**3**_**PO**_**4**_**+HA20%**	2.36±0.18	100±6	3.6±0.10
**H**_**3**_**PO**_**4**_**+HA40%**	2.57±0.23	128±6	3.8±0.14
**H**_**3**_**PO**_**4**_**+HA60%**	2.66±0.20	147±8	3.7±0.13

PVA, polyvinyl alcohol group; H_3_PO_4_, phosphoric acid group.

**Fig 4:**
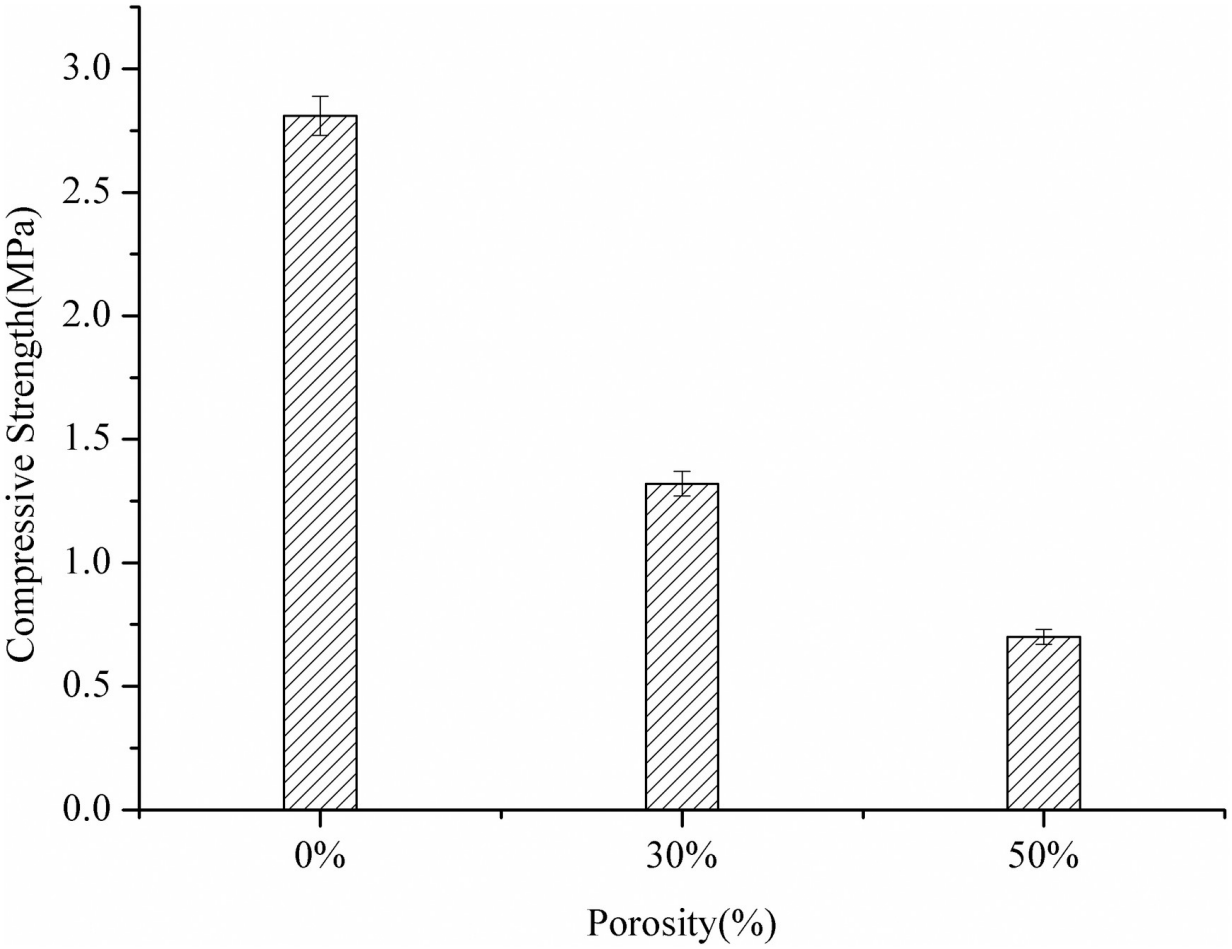
Compressive strength of the scaffolds with different porosity.

The mechanical properties of the scaffolds with different porosities were shown in Fig 4. By increasing the scaffolds’ porosity, the compressive strength significantly decreased, which shows that the scaffold’s porosity has a significant impact on the scaffold’s compressive strength. This result is consistent with that reported by J.C. Le Huec [21]. The increase of the scaffolds’ porosity results in the decrease in the quantity of solid material present in each scaffold. However, the solid material supports the applied load in case the scaffolds loses stability.

In this study, with the phosphoric acid scaffolds, the compressive strength of BCP scaffolds was approximately 2.6 MPa. The tangent modulus was in the range between 100 and 147 MPa. However, with the polyvinyl alcohol scaffolds, the compressive strength of the BCP scaffolds is around 1.34 MPa. The tangent modulus was in the range between 38 and 70 MPa. Both the phosphoric acid scaffolds’ and the polyvinyl alcohol scaffolds’ compressive strength increases with the increase of the content of HA.

Apparently, composition and binder have a significant effect on the scaffolds’ strength. In both groups, as the content of β-TCP increases, the scaffolds’ compressive strength decreases. Guo et al. made the conclusion that the scaffolds’ mechanical properties decrease as the content of β-TCP increases, similar to the findings of this study. [22] Different binders also produce a different influence on the scaffolds’ compressive strength. There is a significant difference between the phosphoric acid scaffolds and the polyvinyl alcohol scaffolds in terms of mechanical behavior. There are some reasons for this: compared with the phosphoric acid scaffolds, the polyvinyl alcohol has higher viscosity, causing the permeability in the bioceramic particles to be very low; therefore, it cannot be sufficiently bonded to bioceramic particles. Moreover, there is no chemical reaction between PVA and powder as the particles are held together by adhesive action of the binder. [20] The binding mechanism between PVA and powder is physical. Calcium phosphate powder is bound by phosphoric acid through a dissolution-precipitation reaction. [23, 24] The binding mechanism of phosphoric acid and powder is chemical. Therefore, the different binding mechanism (physical and chemical) leads to a significant difference between the phosphoric acid scaffolds and the polyvinyl alcohol scaffolds in terms of mechanical properties.

What is more, the scaffolds’ compressive strength should be similar to the strength of the natural bone for which it is being substituted. The compressive strength of the cancellous bone is 2–12 MPa, [25] and the phosphoric acid scaffolds are closer to cancellous bone.

#### 3.3.3 Microstructures of BCP scaffolds

**Fig 5:**
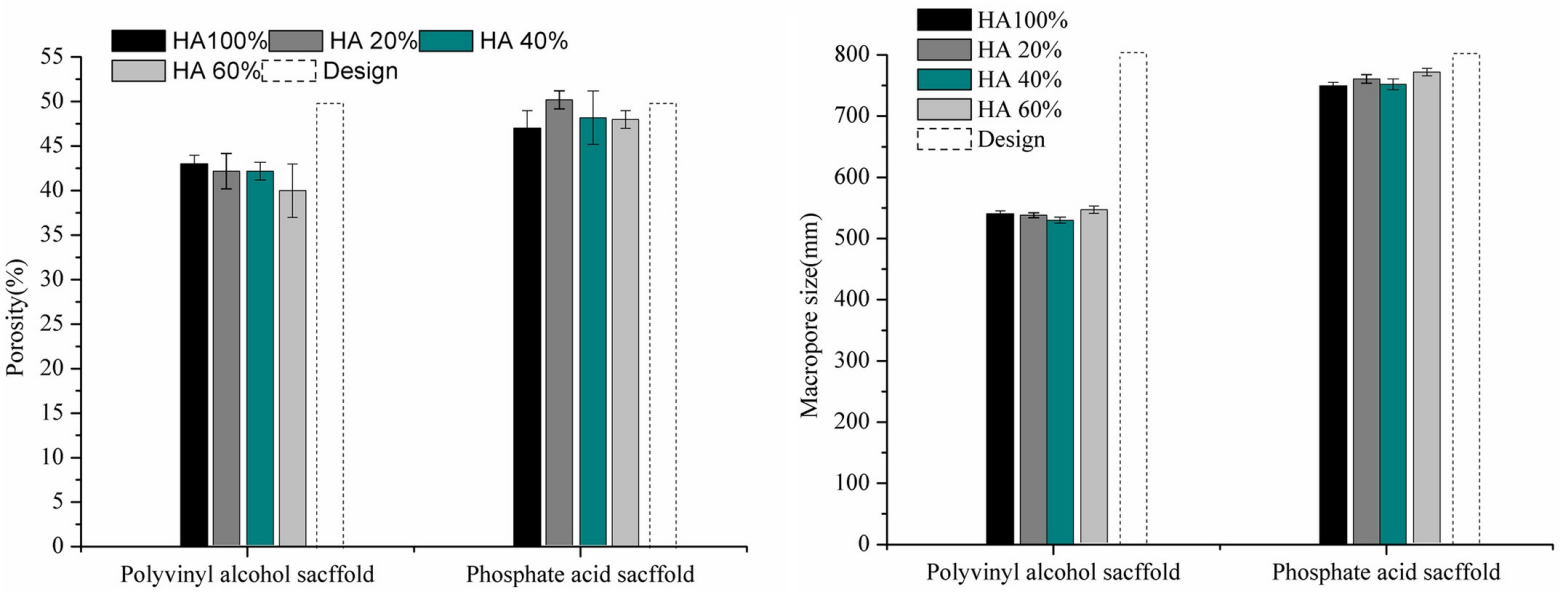
(A) Comparison of experimental porosity. Design porosity was 50% and is represented by dashed line bars. (B) Comparison of experimental macropore size. Macropore size was 800μm and is represented by dashed line bars.

The porosity of the scaffolds shown in Table 5 and measured using two different methods has some difference, which may result from not taking the binder’ density into consideration while calculating the scaffolds’ theory density. Therefore, porosity was also measured by mercury porosimeter as the practical porosity of the scaffolds. The scaffolds’ porosity was analyzed to evaluate the deviations of the printed samples from the design porosity. This information plays an important role in the production of tailored implants. The porosity of the scaffolds is represented in Fig 5A. The porosity of the scaffolds printed is not in accordance with the design porosity, obviously. Composition has no effect on the porosity of the scaffolds printed in both groups. As to the polyvinyl alcohol scaffolds, the porosity of the printed scaffolds is significantly below the design porosity. However, for the phosphoric acid scaffolds, the porosity of the printed specimens presents a deviation of less than 5%. The results demonstrated that the binder has a significant influence on the porosity accuracy.

**Table 5:** Porosity of BCP scaffolds.

Group	Porosity measured by density (%)	Porosity measured by mercury porosimeter (%)
**PVA+HA100%**	42±2	43±1
**PVA+HA20%**	44±2	42±2
**PVA+HA40%**	43±3	42±1
**PVA+HA60%**	42±1	40±3
**H**_**3**_**PO**_**4**_**+HA100%**	48±2	47±2
**H**_**3**_**PO**_**4**_**+HA20%**	49±3	50±1
**H**_**3**_**PO**_**4**_**+HA40%**	47±2	48±3
**H**_**3**_**PO**_**4**_**+HA60%**	49±3	48±1

**Fig 6:**
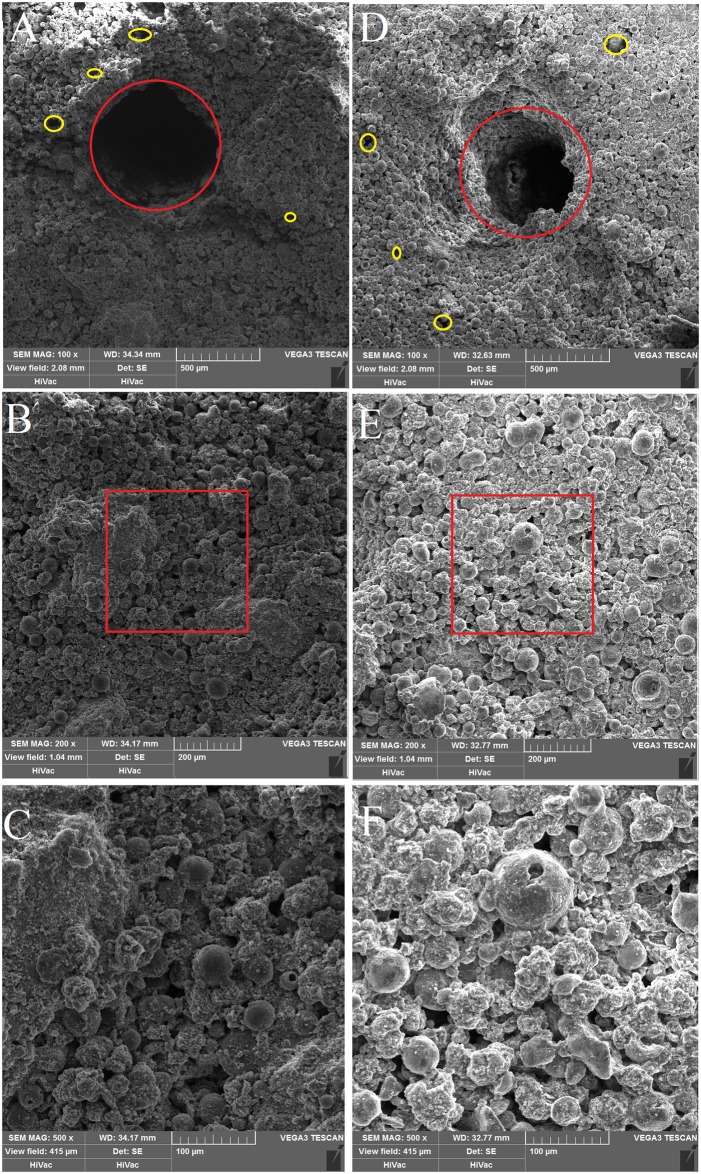
SEM of 3D fabricating bone scaffolds. (A) SEM of the phosphoric acid scaffold. (B) SEM of the phosphoric acid scaffold. (C) High magnification (500 ×) images of the red rectangle in B. (D) SEM of the polyvinyl alcohol scaffold. (E) SEM of the polyvinyl alcohol scaffold. (F) High magnification (500 ×) images of the red rectangle in E. In A, and D, red circles indicate design porosity; yellow circles indicate the tiny gap between the powder particles.

The morphology of the BCP scaffolds was analyzed by SEM images at different magnifications. In the phosphoric acid scaffolds, the fabricated scaffolds is porous. HA powders blend evenly with β-TCP particles and bond with each other, enhancing the adhesive strength of the scaffolds. The practical macropore diameter is similar to the design macropore diameter (Fig 6A). Fig 6B and 6C show the microstructure of the phosphoric acid scaffolds at × 200 and 500 magnification, respectively. Small pores (2~30 μm) are formed between the HA and β-TCP powders, and depend on the pore size of HA and β-TCP particles. The image taken at × 100 magnification of the polyvinyl alcohol scaffolds is represented in Fig 6D, and the morphology is very similar to those of the phosphoric acid scaffolds at the same magnification. However, the practical macropore size is lower than the design macropore size. The macropore are blocked by the unbonded powders. Fig 6E and 6F are images of the polyvinyl alcohol scaffolds taken at × 200 and 500 magnification, respectively. HA spherulites are connected to β-TCP sheets, which are evenly dispersed in the HA microspheric powders, forming small pores of 1~30 μm. The small pore size is similar to those of the phosphoric acid scaffolds because both scaffolds uses the same powder. There were no differences in pore size between the phosphoric acid scaffolds and the polyvinyl alcohol scaffolds with different HA/β-TCP ratios as observed under SEM since the size of HA powder and β-TCP powder are similar.

The influence of the binder and composition on the macropore size is shown in Fig 5B. Though the phosphoric acid scaffolds have no significant decrease in macropore size, and the H6040 scaffolds shows the lowest deviation of macropore size. Apparently, the polyvinyl alcohol scaffolds shows a significant decrease in macropore size compared to the scaffolds design. In terms of the deviation of the macropore size, there is an obvious difference between the phosphoric acid scaffolds and the polyvinyl alcohol scaffolds. Elke Vorndran drew the conclusion that the use of polymeric binders can impair both resolution and compressive strength. [26] This was in accord with the present findings, as phosphoric acid scaffolds possessed high printing resolution. This result revealed that manufacturing methods and binder play vital roles in printing resolution of macropore size.

The scaffolds display two classes of pores. The first class is the design macrospore. The second class is the micropore formed between the materials. That macropores can be connected via microspores. In addition, the scaffolds’ porosity was measured by an AutoPore IV 9500 mercury porosimeter. The principle of mercury porosimeter is Hg intrusion into the pores of the scaffolds. According to the volume of Hg intrusion into the pores of the scaffolds, the porosity of the scaffolds can be calculated. Therefore, from this perspective, it is concluded that the scaffolds possess interconnected pores. For the continuous ingrowth of bone tissue, interconnected porosity is crucial. Open and interconnected pores aid in the transportation of nutrients to the inner parts of a scaffold in order to facilitate cell ingrowth and vascularization. [27, 28]

Based on these results, the binder seem to have an obvious influence on macropore dimensional precision.

For both the phosphoric acid scaffolds and the polyvinyl alcohol scaffolds, the porosity and macropore size of the printed scaffolds showed a significant decrease compared to the scaffolds design. In addition, the porosity of the bone scaffolds was composed of two parts: design porosity, and the tiny gap between the powder material particles. Note that the porosity of the bone scaffolds is composed of two parts: the measured porosity should be higher than the design porosity. On the contrary, the measured porosity was lower than the design porosity, and this is because it is difficult to remove the loose powders from the interconnected pores inside the parts of the scaffolds. Existing loose powders decrease the porosity and the macropore size, because loose powders will be bonded during the post hardening process. This problem is magnified for parts that have small pores, in particular those smaller than 600 μm. [29] In addition, Yoon, J. Y. S. and Castilho, M. regarded the need to remove loose powders from porous scaffolds as one disadvantage of powder printing processes. [30, 31] Future studies may focus on this problem.

There is no difference in surface structure between the phosphoric acid scaffolds and the polyvinyl alcohol scaffolds with different HA/β-TCP ratios as observed under SEM. The surface structure of BCP scaffolds is uneven and rough, which could aid cell attachment. A study of the effects of the surface roughness of hydroxyapatite on human bone marrow cell adhesion, proliferation, differentiation and detachment strength has shown that cell adhesion, proliferation and detachment strength were sensitive to surface roughness and increased as the roughness of the scaffolds increased. [32]

### 3.4 In vitro cell activity and infiltration

**Fig 7:**
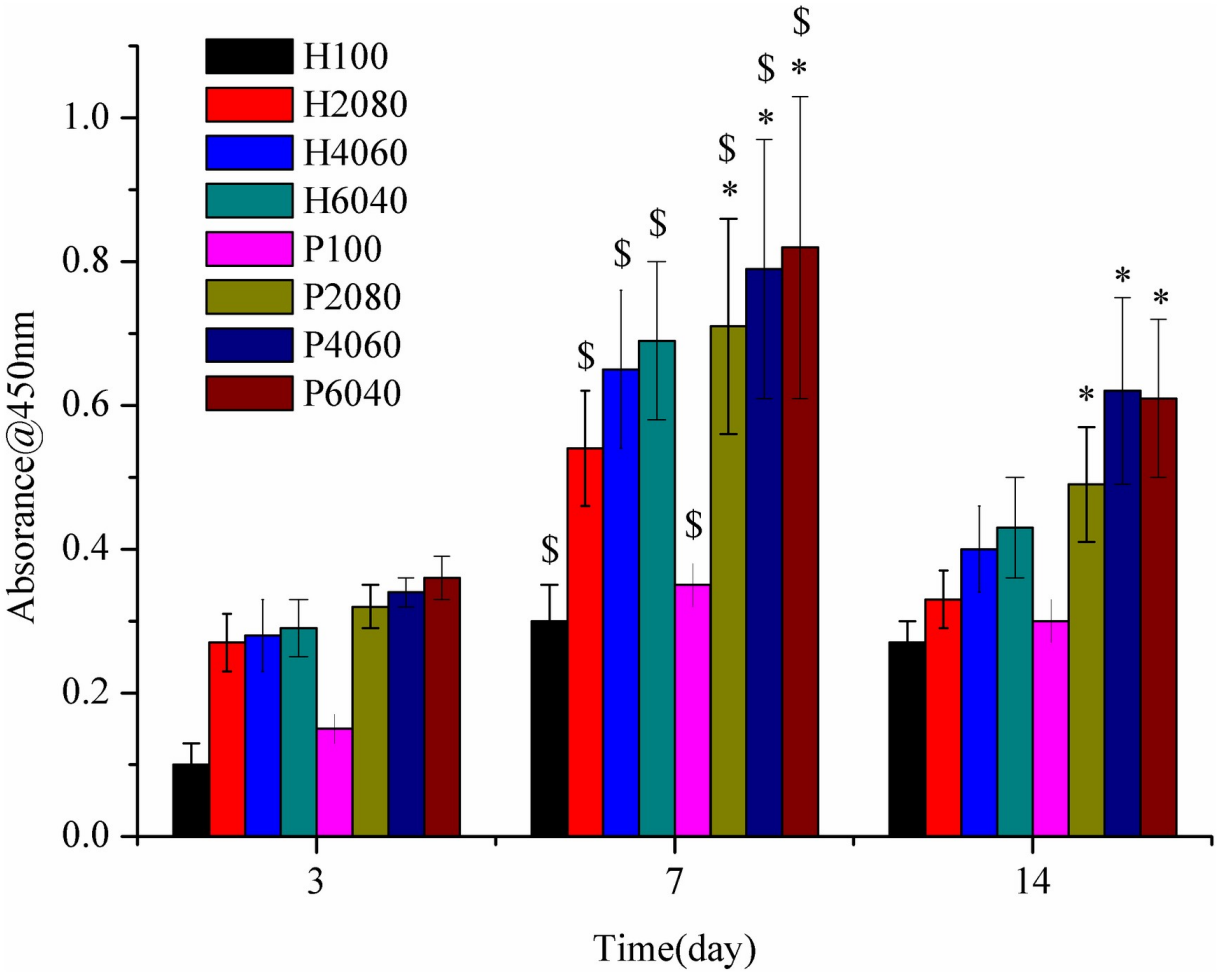
The proliferation of BMSCs cultured on the biphasic calcium phosphate scaffolds for 3, 7, and 14 days. Significantly ($) higher than culture day 3 (p<0.05). *Significantly higher than the scaffolds with H2080, H4060 and H6040 (p<0.05).

The proliferation of the BMSCs cultured on the eight kinds of scaffolds for 3, 7 and 14 days are shown in Fig 7. The analysis shows that the cell proliferation on each scaffold was significantly higher with time. In the first 7 days, the values of absorbance of these eight kinds of scaffolds increased, and they reached the maximum on the 7^th^ day. The values of absorbance dropped on culture day 14 as compared to day 7. The absorbance of H6040 was 122.58%, 27% and 6.15% higher than for the H100, H2080 and H4060 scaffolds on day7, respectively. What’s more, the absorbance of P6040 was 134.28%, 15.49% and 3.79% higher than for the P100, P2080 and P4060 scaffolds on day 7. When the ratios of HA/β-TCP is 60/40 then the phosphoric acid scaffolds’ and the polyvinyl alcohol scaffolds’ absorbance reached maximum. This reveals that the scaffolds with a HA /β-TCP weight ratios of 60:40 is beneficial for cells growth. For the two groups scaffolds, significantly higher proliferation was detected on scaffolds with HA wt% of 20%, 40% and 60% than on the pure HA scaffolds on day3 and day7. This indicates that composition has a significant effect on the proliferation of BMSCs. In addition, as the content of β-TCP increases, the absorbance increases. However, findings were inconsistent with Suzuki T’s report that TCP supports lower rates of proliferation than HA.[33] This may be because some factors (such as pore structure and experimental method) exert a profound effect on the proliferation of BMSCs. The absorbance of the polyvinyl alcohol scaffolds is higher than that of the phosphoric acid scaffolds, which may be due to the better biocompatibility of PVA.

**Fig 8:**
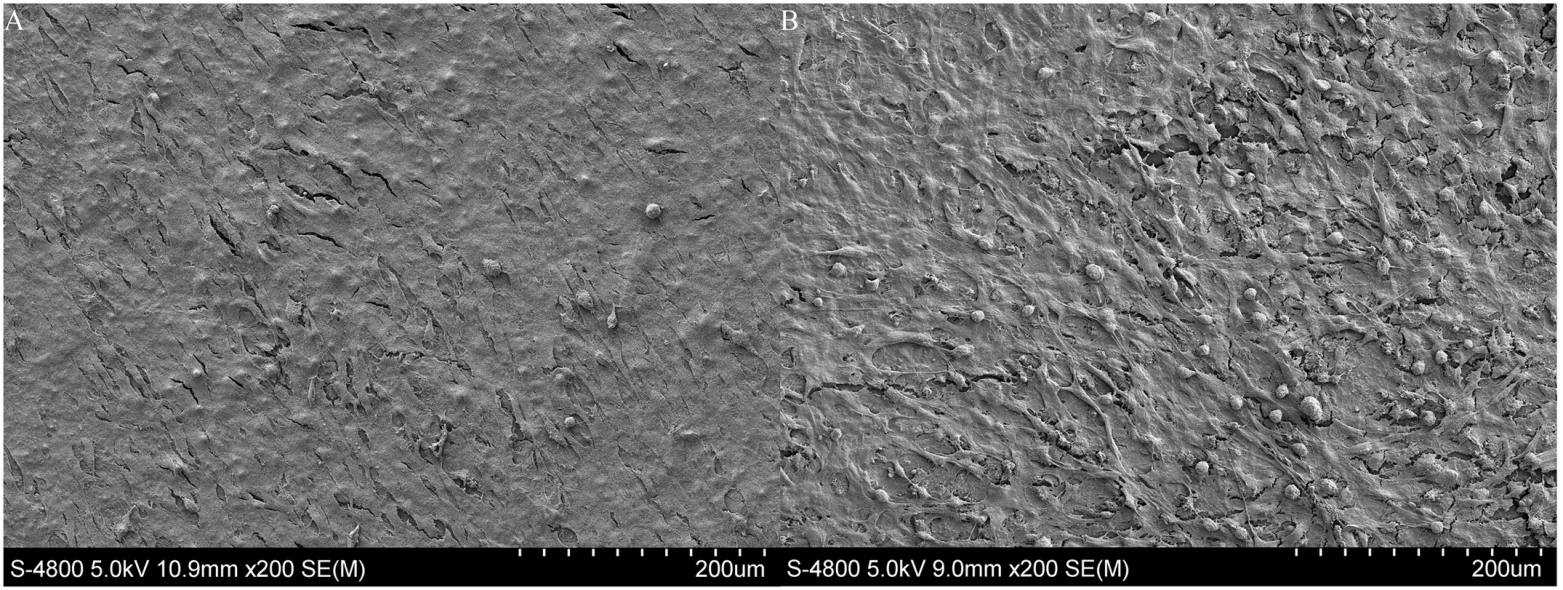
A and B depicts the morphology and distribution of the BMSCs attached on the H6040 and the P6040 scaffolds strut surfaces post culture 14 days.

The attachment and morphology of BMSCs on the scaffolds were observed by SEM (Fig 8A and 8B). After 14 days in culture, BMSCs attached to the surface of the scaffolds presenting well-spread morphology on each type of scaffolds. These SEM images confirmed the biocompatibility of the 3D printed HA/β-TCP scaffolds.

In this study, a continuous increase in the values of absorbance from day 3 to day 7 indicates that cells seeded on the eight kinds of scaffolds supported the growth of the cells. However, a drop in the values of absorbance on culture day 14 compared to day 7 indicates the differentiation of BMSCs into the mature osteoblast phenotype towards the bone lineage.

## 4 Conclusion

Three-dimensional (3D) printing technology has been used successfully in the fabrication of BCP (HA/β-TCP) scaffolds of a desired shape with interconnected pores and controlled porosity. This study demonstrated that the polyvinyl alcohol (PVA) solution concentration of 0.6 wt% with a supplementation of 0.25 wt% Tween 80 improved inkjet printing. Fabrication accuracy, micro-architecture and suitable mechanical properties demonstrated that the phosphoric acid has a better printable performance than polyvinyl alcohol solution binder for 3D printed bone scaffolds. The use of phosphoric acid not only improves the mechanical properties of the scaffolds, but also maintains the design porosity of the scaffolds. Different composition ratios of HA and β-TCP had a slight influence on the mechanical properties. As the weight ratio of HA to β-TCP in the scaffolds increased, the scaffolds’ compressive strength increased. SEM showed that the size of HA and β-TCP particles determined the micropore size. Manufacturing methods and binder played vital roles in printing macropore size with resolution. Both the polyvinyl alcohol scaffolds and the phosphoric acid scaffolds showed good cellular affinity permitting attachment of BMSCs to the material surfaces and supporting the appropriate proliferation and functions of BMSCs. However, the polyvinyl alcohol scaffolds displayed higher dimensional variations encountering with water solution, which revealed that the shape of scaffolds deformation is larger than 5%. Considering the scaffolds’ mechanical and biocompatible properties, the phosphoric acid scaffolds with a HA /β-TCP weight ratios of 60:40 may be the best candidate for bone tissue engineering applications. Future studies may investigate *in vivo* the biological behavior of phosphoric acid scaffold with HA /β-TCP weight ratios of 60:40.
